# Agroecosystem specific climate vulnerability analysis: application of the livelihood vulnerability index to a tropical highland region

**DOI:** 10.1007/s11027-014-9568-1

**Published:** 2014-07-04

**Authors:** Belay Simane, Benjamin F. Zaitchik, Jeremy D. Foltz

**Affiliations:** 1 College of Development Studies, Addis Ababa University, Addis Ababa, Ethiopia; 2 Department of Earth and Planetary Sciences, Johns Hopkins University, Baltimore, MD 21218 USA; 3 Agricultural and Applied Economics, University of Wisconsin-Madison, Madison, WI 53706 USA

**Keywords:** Vulnerability, Adaptive capacity, Exposure, Climate change, Agroecosystem

## Abstract

In topographically diverse highland terrain, socio-economic and environmental conditions can vary dramatically over relatively short distances. This presents a challenge for climate resilient development strategies, as exposure to climate variability and change, climate impacts, and adaptive capacity differ between communities located within common cultural and administrative units. The Livelihood Vulnerability Index (LVI) framed within the United Nations Intergovernmental Panel on Climate Change (IPCC) vulnerability framework (LVI-IPCC) offers a tool to assess climate vulnerability through direct household surveys. This makes it particularly appropriate for analyses at sub-community and community scales. Here we apply the LVI-IPCC to communities of Choke Mountain, located in the Blue Nile Highlands of Ethiopia. Recognizing the physiographic and climatic diversity that exists in this mountainous environment, we implement LVI-IPCC analysis for 793 mixed crop-livestock farming households using the five distinct agroecological systems (AES) that compose the populated area of Choke Mountain as a framework for analysis. For each AES, an LVI index, adaptive capacity metric, and LVI-IPCC vulnerability score was calculated. We found that each of these metrics varied systematically across AES. High elevation sloping lands and low elevation steep lands exhibited relatively low adaptive capacity and high vulnerability while midland AES had higher capacity and lower vulnerability. These results suggest that resilience building interventions for Choke Mountain ecosystems should be targeted to address the specific circumstances of each AES. The approach of applying LVI-IPCC at AES scale could be applicable to other climate vulnerable mountainous regions.

## Introduction

Ethiopia is frequently identified as a country that is highly vulnerable to climate variability and change (Parry et al. 2007; WorldBank 2010; Conway and Schipper 2011). At the national scale, vulnerability to climate is evident in economic and social sensitivity to interannual precipitation variability and extreme climate events, particularly drought (WorldBank 2011). These sensitivities are a product of large interannual climate variability and an economy that is highly dependent on agriculture (Byerlee et al. 2007) as well as institutional factors that can create socio-economic crises even in the absence of a large meteorological anomaly (Smakhtin and Schipper 2008). The combination of sensitivity to historic climate variability, limited capacity at institutional levels, and projections of significant future climate change are cause for concern that Ethiopia will be strongly and negatively impacted by changing climate patterns in the coming decades (McSweeney et al. 2010a, b; Ethiopian EPA 2011).

However, aggregated national statistics do not capture the complex distribution of vulnerabilities that is present at the local level. This is particularly true in the steeply dissected highland region that is home to a majority of the Ethiopian population. In this mountainous terrain, prevailing climate conditions and the sensitivity of agricultural systems to climate variability can change over a distance of even a few kilometers. Dramatic spatial heterogeneity is also seen in soil qualities, steepness of slope, and access to infrastructure to support transport, agricultural technologies, and capacity building. As a result, an estimate of vulnerability at the national, state or even district scale is inadequate to capture the full range of climate vulnerabilities within the Ethiopian highlands or to design effective resilience building interventions.

It should be noted that vulnerability in this context has been defined in a number of different ways (Gallopín 2006; Füssel 2007), and the outcome of any effort to quantify vulnerability will be sensitive to the choice of definition and metrics. Here we accept the general description of Kelly and Adger (2000), that vulnerability is the ability or inability of individuals or social groupings to respond to, in the sense of cope with, recover from, or adapt to, any external stress placed on their livelihoods and well-being. In the context of climate change, the Intergovernmental Panel on Climate Change (IPCC) adopts a variant of this definition, stated as “the degree to which a system is susceptible to, or unable to cope with, adverse effects of climate change, including climate variability and extremes” (IPCC 2007). In this definition, vulnerability is typically presented as a condition of three inter-related factors: exposure to impacts, sensitivity to impacts, and capacity to adapt to impacts (Snover et al. 2007).

The IPCC definition of vulnerability does not specify any particular assessment methodology or scale of analysis. One useful methodology for applying the definition at household and community levels is the Livelihood Vulnerability Index (LVI) adapted to the IPCC framework (LVI-IPCC; Hahn et al. 2009). The LVI-IPCC is an implementation of the Sustainable Livelihoods Approach to development analysis (Chambers and Conway 1992), according to which communities are described in terms of their natural capital, social capital, financial capital, physical capital, and human capital. Stresses related to climate change map onto each of these capitals, but climate exposures and adaptation actions are typically not explicitly considered in the approach. The LVI reorganizes the Sustainability Livelihoods Approach into new categories, which includes an explicit climate component, and is framed in a manner amenable to the use of household survey data. The LVI-IPCC, also developed by Hahn et al. (2009), maps the LVI components onto the three IPCC contributing factors to vulnerability—exposure, adaptive capacity, and sensitivity.

In the first implementation of LVI-IPCC, Hahn et al. (2009) applied the methodology to two villages in Mozambique with differing socio-economic and environmental conditions. They found that one village was more constrained by a physical limitation—water resources—while the other had more severe socio-demographic vulnerabilities. The LVI-IPCC methodology has since been applied to study communities and regions in other parts of the developing world. Mohan and Sinha (2010), for example, applied the LVI-IPCC across districts in the Ganges River basin and found significant differences in vulnerability at a district level. Differences between districts were attributed to a number of factors, including relative degree of urbanization: more highly urbanized districts had greater adaptive capacity and therefore lower climate vulnerability. In a study of climate vulnerability in Northern Ghana, Entwire et al. (2013) applied the LVI-IPCC and found regional scale discrepancies in vulnerability, based in large part on access to water resources.

Climatically diverse regions, including the Ethiopian Highlands and many other tropical highland regions, present a challenge for policy-relevant implementation of the LVI-IPCC, simply because climatic and biophysical conditions change so dramatically over short distances. A critical question, and one that has not been addressed in the LVI-IPCC literature to date, is how to select a unit of aggregation that captures diversity across a climatically heterogeneous area while being generalizable enough to inform adaptation strategies beyond the specific communities surveyed in the study. Here, we use the agricultural ecosystem (AES) as the unit of analysis. This decision was made because the AES represents the intersection of (1) a set of agriculturally relevant climatic factors—i.e., the agroecological zone—with (2) soils and physiographic variables relevant to crop production, and (3) a prevailing set of cropping practices. As such, the AES is a relevant target for designing agriculture-oriented climate resilience strategies, and is therefore a critical unit for climate vulnerability analysis.

Applying an AES-based generalization to household-level analysis allows us to map vulnerability profiles across the landscape. This ability to generalize is critically important for adaptation planning, both because it makes it possible to exchange learning experiences across communities with similar vulnerability profiles and because it allows decision makers with broad geographic jurisdictions to understand patterns of vulnerability across relatively large areas.

## Choke Mountain

**Fig. 1:**
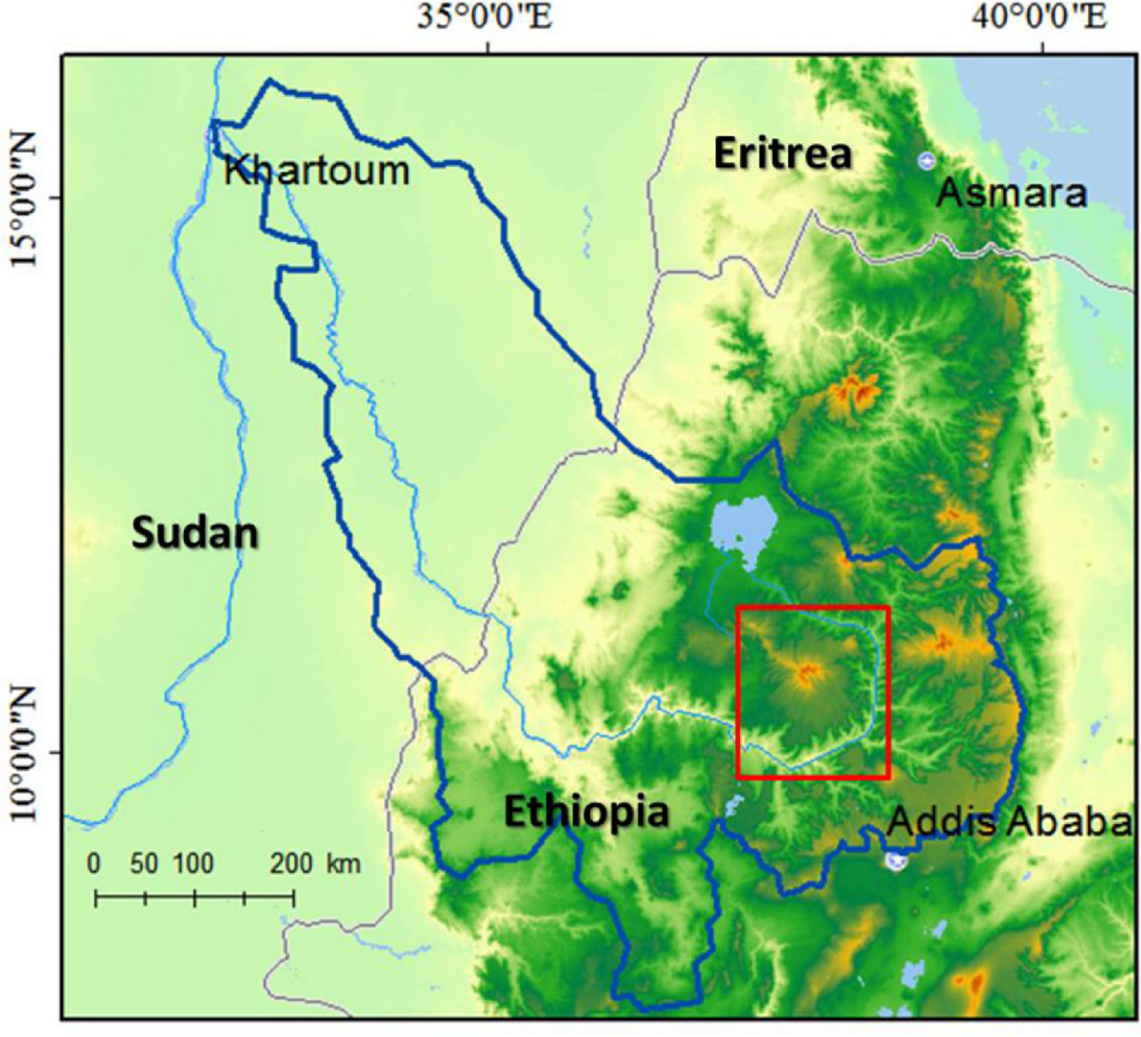
Choke Mountain (*red box*) and the surrounding Blue Nile River basin (*blue line*)

Choke Mountain is located in the Blue Nile (Abay) Highlands region of Ethiopia (Fig. 1). The mountain’s peak elevation is over 4,000 m, but the edge of the mountain is defined in three directions by the Blue Nile gorge, where elevation drops to below 1,000 m. Over a distance of less than 70 km, then, one finds hot, dry valleys, gently rolling, deep soiled midland plains, and cool, wet alpine zones. Complex topography creates strong local gradients in precipitation, temperature and soil properties, although rains are characteristically intense and erosive across topographic zones, and soils are deeply weathered and erodible over most of the mountain. The landscape is dominated by low-input mixed crop-livestock subsistence agriculture, with cultivation extending from the Blue Nile Gorge up to nearly the mountain’s summit. The dominant crops are tef (*Eragrostis tef*), maize (*Zea mays*) and wheat (*Triticum aestivum and T. Durum*), while barley (*Hordeum vulgare*), potato (*Solanum tubererosum*), fava beans (*Phaseolus vulgaris*), chickpeas (*Cicer arietinum*), oat (*Avena sativa*), and sorghum (*Sorhum bicolor* Moench) are also grown in some parts of the mountain. Historically, the alpine zone surrounding Choke Mountain summit was covered in forest and natural grass- and shrub-lands, but increasing population and associated deforestation and land degradation have helped push the extent of cultivation to as high as 3,800 m elevation (Simane 2011).

Multiple sources of pressures on Choke ecosystems have taken a toll on the region’s natural resources, with each posing its own management challenge. First, the natural resources base is under intense pressure from population growth and erosion-inducing traditional farming and management practices. Second, farming communities face severe constraints related to intensive cultivation, overgrazing and deforestation, soil erosion and soil fertility decline, water scarcity, and demand for livestock feed, and fuel wood. Third, climate change may already be contributing to these challenges. There has been a perceived increase in extreme rain events, and regional temperatures have exhibited an upward trend over the past 20 years (Simane et al. 2012). Together, these challenges have contributed to a documented decline in yields in some areas, and portions of the mountain have deteriorated from food surplus to food deficit areas within the past 20 years.

The Choke Mountain ecosystem is densely populated, with an average of 260–270 people per km^2^ (Simane 2011). Livelihoods are primarily dependent on subsistence mixed crop-livestock agricultural systems, with very low inputs and outputs. There is no significant natural forest cover. The major natural habitats are moist moorland, sparsely covered with *Giant Lobelia* spp., *Alchemilla* spp., *Festuca* spp. and other grasses. Settlements are common up to about 3,600 m elevation, and extensive agricultural activity is found on steep slopes as well as on almost all gentle and moderately sloped land.

Under undisturbed conditions, soils on Choke Mountain tend to be deep—natural depths are thought to be more than 50 cm over most of the BNH, with rooting depths up to one meter, allowing for local variability across terrain. These deep, weathered tropical soils are highly susceptible to erosion, and on lands cultivated using traditional methods the rate of soil loss can exceed the rate of soil generation by a factor of 4 to 10 (Hurni 1988). The process is exacerbated in large part by the prevalence of traditional criss-cross ox-drawn tillage systems that promote rapid erosion (Nyssen et al. 2000).

There is very little woody plant cover. *Erica spp* and *Hypericum revolutum* are found in patches. *Arundinaria alpine* is found both as a homestead plantation as well as part of the natural vegetation cover in the area, albeit very sparsely. *Erithrina brucei* is cultivated as a border demarcation plant in the area. *Eucalyptus globulus* is extensively gown as a plantation, and some of the residents of the area have become dependent on it for their livelihood.

Nutrient depletion is a serious problem in the region. Average soil loss rates on croplands have been estimated at 42 t ha^−1^ yr^−1^ but may reach 300 t ha^−1^ yr^−1^ in individual fields (Hurni 1998). High rates of on-field erosion are particularly problematic given that nutrients in Choke soils tend to be concentrated in the upper portion of the soil column. At present, soil acidity in the Choke is one of the major causes for land degradation. Nitosols and Acrisols that compose about 26 % of the area are acidic, with pH < 5. At harvest, both grain and straw is removed from the field with no residue returned to the soil. The use of animal dung and crop residues as substitutes for household energy have further contributed to reduced soil fertility and agricultural productivity in the area. Nevertheless, the depth of the soil profile means that there are still significant soil resources, and experience indicates that productivity can be maintained and enhanced through effective field scale and landscape scale sustainable land management practices (Simane et al. 2012).

**Fig. 2:**
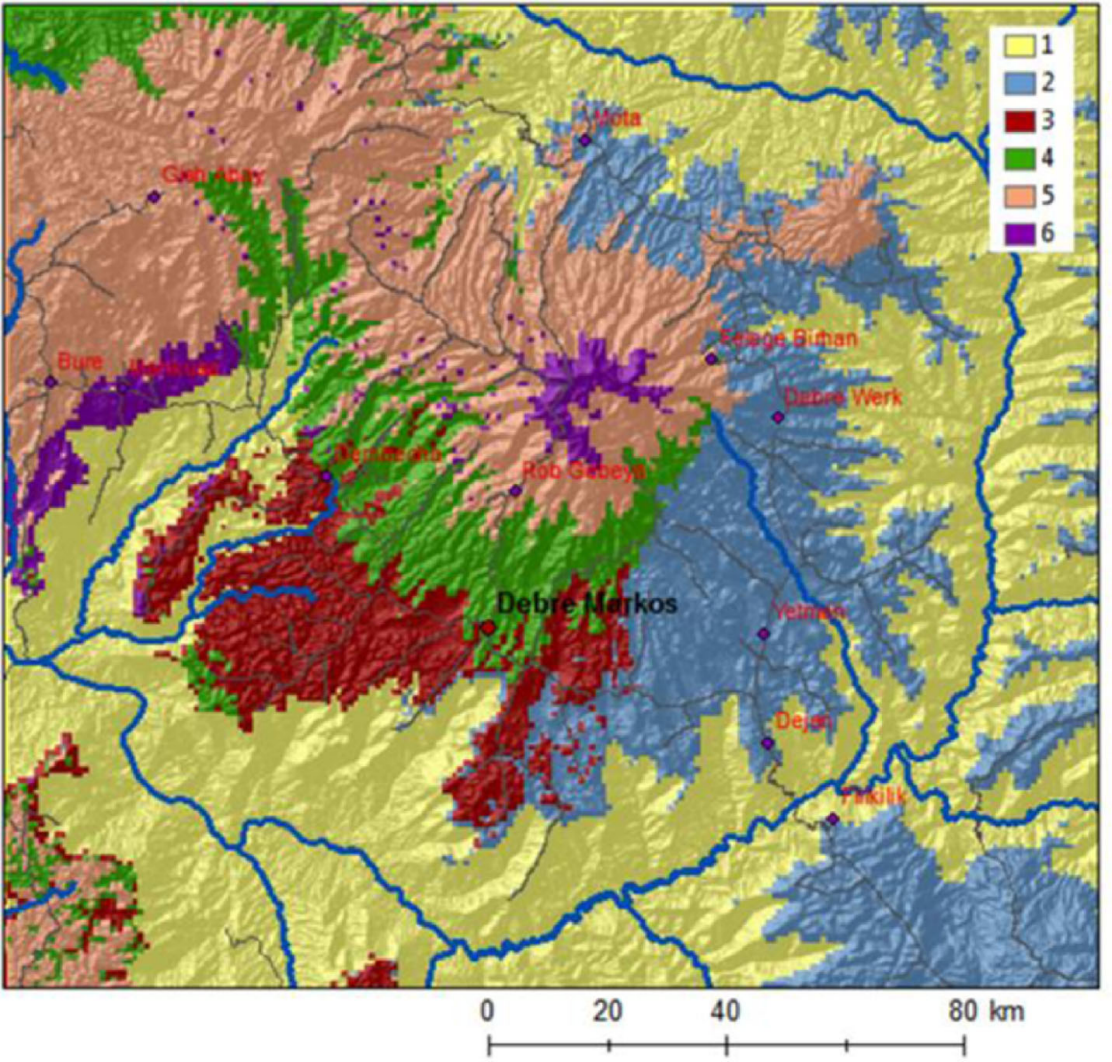
The six major agroecosystems of Choke Mountain: lowland and valley fragmented agroecosystems (AES 1; total area 7,200 km^2^), midland plains with black soil (AES 2; 3,200 km^2^), midland plains with brown soils (AES 3; 1,600 km^2^), midland sloping lands (AES4; 1,300 km^2^), hilly and mountainous highlands (AES5; 2,400 km^2^), and Afro-alpine (AES6; 250 km^2^). Figure based on analysis from Simane et al. (2013)

Considering physical, agricultural, and socio-economic factors, Simane et al. (2013) classified the Choke Mountain watersheds into six major agroecosystems (AES) using field-based agroecosystem analysis (Conway 1985) in combination with automated landscape classification (Fig. 2). The definition of AES was based on the overlay of three inputs: an agro-climatic zoning based on precipitation and temperature, a soil and terrain analysis, and a map of the distribution of farming systems. Simane et al. (2013) demonstrate that the AES show a remarkable degree of differentiation in terms of their defining constraints, opportunities, production orientation, and socio-economic characteristics of farmers. The classification provides a lens for vulnerability and adaptation analysis that considers the geographical differentiations as well as the socio-economic stratification of the agricultural sector of the study area. We note that only AES 1–5 are inhabited to any significant extent. AES 6 is the mountain summit, characterized by natural vegetation that is used commonly for grazing and as source fuel and construction wood. As there is no significant permanent population in AES 6 it is not included in LVI-IPCC analysis in this paper.

Observational studies have shown steady warming in Ethiopia since 1960, at a rate of ~0.28 °C per decade (Conway and Schipper 2011). Climate models project continued warming for Ethiopia through the end of the 21st century, with warming over the 20th century baseline of 0.7–2.3 °C for the 2020s, 1.4–2.9 °C for the 2050s, and 1.5–5.1 °C for the 2090s, depending on global greenhouse gas emissions trajectories and the climate model used in the projection (Conway and Schipper 2011). Projections for precipitation are far more uncertain, both for Ethiopia in general (Conway and Schipper 2011) and for the Blue Nile Highlands specifically (Zaitchik et al. 2012). Studies that report the ensemble average of global climate model projections for all models participating in IPCC intercomparisons generally project an increase in precipitation by the end of the 21st century, but the spread between models encompasses projections for both significant increase and substantial decrease in precipitation in the western part of the country.

Furthermore, changes in total precipitation might be less important to livelihoods in the region than changes in intensity, timing, and variability in precipitation. For example, some analyses project that the proportion of rainfall that falls in heavy precipitation events will increase throughout Ethiopia, with a particularly strong tendency during the July to September rains that affect Choke Mountain (McSweeney et al. 2010a; b, Ethiopian EPA 2011). Both the frequency and intensity of droughts in Ethiopia have increased recently and inflicted severe damage to the livelihoods of millions of people. At the same time, increases in floods have stressed social institutions and intensified the vulnerability of households. A recent World Bank study projects that climate change will reduce Ethiopia’s gross domestic product (GDP) growth by between 0.5 and 2.5 % each year unless effective steps to build resilience are taken (WorldBank 2010).

## Methodology

### Defining vulnerability profiles and indicators for Choke Mountain

Hahn et al. (2009) developed and demonstrated the LVI and LVI-IPCC in an application to two villages in Mozambique. For their application, they defined seven major components of vulnerability that map onto the three IPCC contributing factors to vulnerability (exposure, adaptive capacity, and sensitivity). We adopt this framework for Choke Mountain but with some modification to the major components in order to match the conditions and constraints facing agricultural households in the Choke region. In place of the seven major components of Hahn et al. (2009) we define eight “profiles” related to vulnerability: climate, ecosystem, agriculture, wealth, technology, infrastructure, community, and social.

Each profile is defined by a set of indicators, and each has a hypothesized relationship with climate vulnerability. The indicators used here (Table 1) were developed based on a review of previous LVI studies in rural Africa and our earlier experience in the region. The definition of these geographically and culturally customized indicators is an important part of the LVI process. For Choke, it was important to consider changes in climate extremes along with changes in average temperature and precipitation, and it was also necessary to include indicators on ecosystems and landscape in addition to indicators on agricultural practices. For social capital, it was informative to include participation in existing community organizations and sustainable land management projects, as a large number of such organizations and projects are already active in the region. Under human capital, the sex of household head was deemed relevant because the cultural norm in the region is for female heads of households to rent their land rather than plowing it themselves; hence, the sex of head of household has a direct impact on the household’s ability to utilize its property resources on account of plowing traditions, even though there is no inherent reason that sex of household head should impact human capital.

**Table 1:** Vulnerability factors, livelihood capitals, profiles, and indicators used for LVI analysis using the IPCC framework

Vulnerability factors	Livelihood capitals	Profiles	Indicators	Units	Hypothesized functional relationship
Exposure		1. Climate	• Change in temperature • Change in precipitation • Occurrence of extreme events	Changes over time, °C Changes over time, mm No of events over the last 20 years	Larger change or frequency = higher exposure
Sensitivity	Natural capital	2. Ecosystem	• Land suitability for agriculture • Sustainability of land use system • Land cover change (primarily deforestation/reforestation) • Use of soil water conservation techniques) • Irrigation potential	Avg. scale values of soil depth, terrain, drainage, and fertility f (1–5) Assumed intensity of management (High, Medium and Low) % change over the baseline % of land with SWC structures Ha of land suitable for irrigation	More forest cover, suitable land, and access to irrigation = lower sensitivity
		3. Agriculture	• Annual total production (inverse) • Changes in productivity • Diversity of crop species	Tons of total product harvested Yield in tons/ha Number of crops in the system	Greater productivity and diversity = lower sensitivity
Adaptive capacity	Financial capital	4. Wealth	• Farm size • Number of livestock • Savings at household level • Existing loans • Non-agricultural income	Ha/HH TLU/HH Amount of Birr (local currency)/HH Amount of Birr/HH Amount of Cash obtained per year	Greater wealth = greater adaptive capacity
	Physical capital	5. Technology	• Insecticide and pesticide supply • Fertilizer supply • Improved seed supply • Irrigation potential	% of HHs using insecticide % of HHs applying fertilizer % of HHs using improved seed % of HHs practicing irrigation	Better access to technology = greater adaptive capacity
		6. Infrastructure	• Access to all-weather roads • Access to schools • Access to veterinary services • Access to markets • Access to savings and credit • Access to electricity • Access to telephone	Walking distance in hours Walking distance in hours Walking distance in hours Walking distance in hours % of HHs using credit % of HHs accessing lights % of HHs using telephone	Better access to infrastructure = greater adaptive capacity
	Human capital	7. Community	• Sex of household head • Education level • Availability of extension • Skills/training • Health services • Radio ownership	Male/Female % of HH heads No of Das/village No of training HH head attended Walking distance in hours % of HHs who have radio	More human capital, information and services = greater adaptive capacity
	Social capital	8. Social	• Governance • Membership in CBOs • Participation in projects • Availability of bylaws • Number of non-working days/ month • Tradition of working together	1–5 scale (election of leadership) Yes/No Participation index Yes/No No of days % of HH who have tradition of working together	Fewer non-working days and more tradition of working together = greater adaptive capacity

This table is modeled on a similar table in Hahn et al. (2009), but profiles, indicators, and hypothesized functional relationships are customized for this study

The eight vulnerability profiles were then mapped onto the three IPCC contributing factors to vulnerability in the same manner used in Hahn et al. (2009). Each of the profiles, with the possible exception of climate change, can also be associated with one of the five types of capital used in the Sustainable Livelihoods Approach (Chambers and Conway 1992) (Table 1).

### Household survey

A cross-sectional survey was conducted from April to June, 2012 engaging 793 mixed crop-livestock farming households in 36 communities spread across all 5 agroecosystems (87 in AES1, 183 in AES2, 148 in AES3, 284 in AES4, and 91 in AES5). The sampling method followed a stratified random sampling technique. The survey was conducted by development agents from local agriculture offices who were trained on the selection of households, timing and manner of conducting the survey and data recording. Verbal consent was obtained from each head of household.

The survey questions were designed to address the eight profiles used in calculating the LVI. They included inquiries on socio-economic and environmental attributes as well as questions related to farmers’ perceptions of climate change and adaptation methods. The surveyed farmers were also asked questions about their observations regarding patterns of temperature and rainfall over the past 20 years. Household surveys were paired with biophysical survey to assess indicators related to land suitability and irrigation potential.

### Calculating the LVI

The calculation of LVI follows the method used in Hahn et al. (2009) and Mohan and Sinha (2010). It is a balanced weighted average approach (Sullivan 2002) where each sub-component contributes equally to the overall index even though each major component of different livelihood assets includes a different number of sub-components.

First, each indicator was standardized to a common scale:

1 $$ {I}_v=\frac{I_a-{I}_{min}}{I_{max}-{I}_{min}} $$

where *I*_*v*_ is the standardized value for the indicator, *I*_*a*_ is the value for the indicator *I* for a particular AES *a*, *I*_*min*_ is the minimum value for the indicator across all the AESs, and *I*_*max*_ is the maximum value for the indicator across all the AES. Next, a profile average value was calculated as:

2 $$ {\boldsymbol{P}}_{\boldsymbol{a}}=\frac{{\displaystyle \sum {\boldsymbol{I}}_{\boldsymbol{v}}}}{\boldsymbol{N}} $$

where *P*_*a*_ is the value for the profile in AES *a* and *N* is the number of variables in the profile. Values for each of the eight profiles were then combined to obtain the AES level LVI:

3 $$ LV{I}_a=\frac{{\displaystyle {\sum}_{p=1}^8}{N}_p{P}_{a, p}}{{\displaystyle {\sum}_{p=1}^8}{N}_p} $$

where LVI_a_ is the Livelihood Vulnerability Index for AES a and N_p_ is the number of indicators in each profile.

The eight profiles are combined according to the IPCC categorization scheme as:

4 $$ C{F}_a=\frac{{\displaystyle {\sum}_{p=1}^f}{N}_p{P}_{a, p}}{{\displaystyle {\sum}_{p=1}^f}{N}_p} $$

where *CF*_*a*_ is an IPCC contributing factor (exposure (*E*), sensitivity (*S*), or adaptive capacity (*A*)), *f* is the number of profiles associated with the contributing factor, and *p* is indexed to the profiles associated with the *CF*. Finally, the LVI-IPCC for AES *a* is calculated as:

5 $$ LVI- IPC{C}_a=\left({E}_a-{A}_a\right)\ast {S}_a. $$

The LVI-IPCC is scaled from −1 (least vulnerable) to 1 (most vulnerable) and is best understood as an estimate of the relative vulnerability of compared populations.

## Vulnerability index components

In this section we provide an analysis of the key components used to generate the vulnerability index, highlighting the key components and where they differ between agro-ecosystems. Table 2 provides summary characteristics of the demographic and socioeconomic data for the study population, which falls under the index category of adaptive capacity--human capital. A total of 790 people participated in the study and of those studied, 737 (93 %) were male and 53 (7 %) were female. In terms of age category, the majority of respondents (81 %) are within the active working age group (31–65), while 32 respondents (4 %) are above 65 years of age. Seven hundred and thirty one respondents (94 %) were married and the remainder was single, divorced, or widowed. Education levels are low with many of the respondents (45 %) being illiterate, approximately 52 %, were either able to read and write or had primary education (1–4 years schooling), and only 2.3 % of the respondents having attended secondary school.

**Table 2:** Characteristics of the study population (*n* = 793)

Characteristics	Category	%
Gender	Male headed households	93.3
	Female-headed households	6.7
Age	15–30	15.2
	31–65	80.7
	>65	4.1
Marital status	Married	94.3
	Not married	1.7
	Divorced	4.0
Education	Illiterate	45.1
	Reading and writing	42.2
	Primary school	10.1
	Secondary school	2.7

### Exposure profile: climate change

Active adaptation to climate change requires that farmers first notice that the climate has changed and that they then identify useful adaptation options and implement them (Maddison 2006; Sewagegn 2011). People living in different agro-ecological systems are believed to perceive climate change differently, even when the systems are in close proximity to one another, due to contrasts in local climate impacts, as well as to differing socio-economic perspectives on these impacts (Simane et al. 2012). From our random sample data we are able to assess farmer perceptions of climate change and how they vary across agro-ecosystems, with the results shown in Table 3. These measures fall under the LVI-IPCC category Exposure-Climate.

**Table 3:** Perceptions of climate change over the past 20 years, as reported in household surveys. All values are percent of responses in the given AES

	AES1	AES2	AES3	AES4	AES5	All households
Temperature						
Increasing	95.2	78.3	94.6	79.6	80.2	85.5
Decreasing	2.4	14.1	3.4	18.6	12.1	10.1
No change / constant	2.4	1.6	0.7	0.9	3.3	1.8
I don’t know	0.0	5.4	1.4	0.0	4.4	2.2
Significantly different (95 % level) from AES	2, 4, 5	1, 3	2, 4, 5	1, 3	1, 3	
Precipitation						
Increasing	8.3	13.0	2.7	0.9	5.5	6.1
Decreasing	65.5	56.0	54.7	69.9	60.4	61.3
No change	4.8	1.6	1.4	1.8	1.1	2.1
Change in seasonality	10.7	23.4	31.1	23	27.5	23.1
Increased drought frequency	9.5	3.8	7.4	3.5	3.3	5.5
I don’t know	1.2	1.1	2.7	0.9	2.2	1.6
Significantly different (95 % level) from AES…	3	3	1, 2	None	None	
Extreme events						
Did you experience extreme weather events or climate hazards	78.3	85.0	58.3	85.7	69.0	74.4
Significantly different (95 % level) from AES…	5	5	5	None	1, 2, 3	

**Fig. 3:**
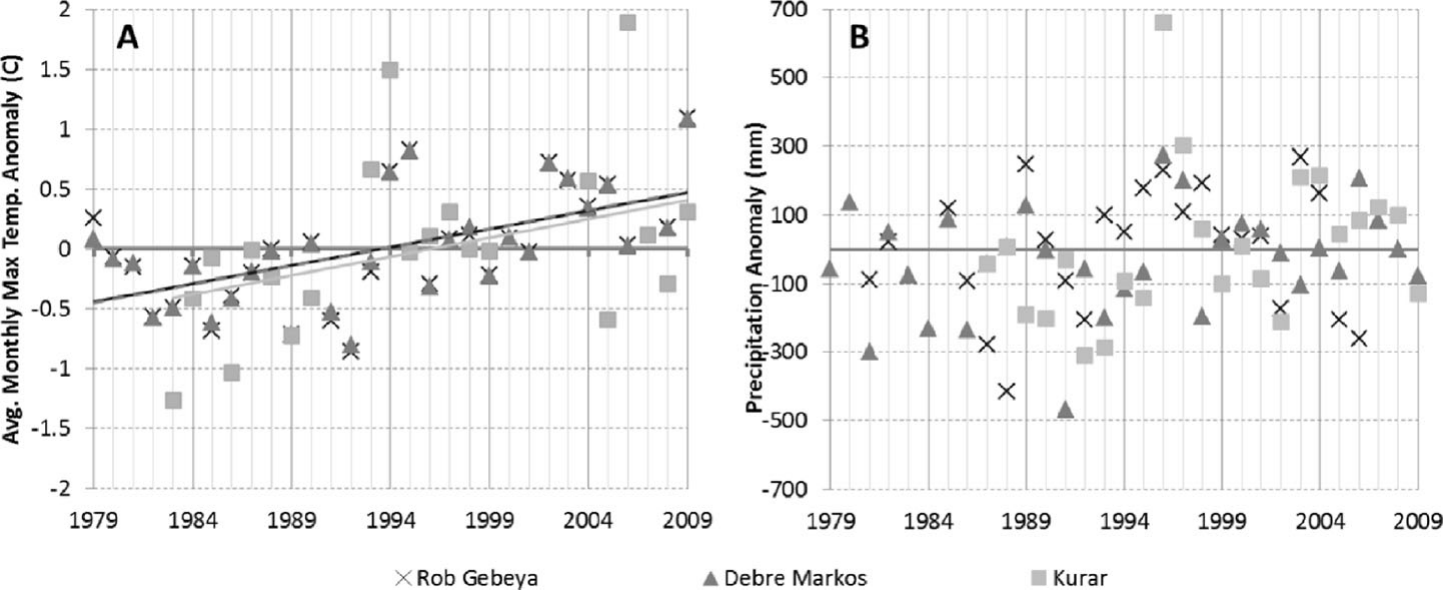
Meteorological station records of (**a**) annually averaged monthly maximum temperature anomaly and (**b**) annual precipitation anomaly for Rob Gebeya (AES5), Debre Markos (AES3/4) and Kurar (AES1). Linear trends in (**a**) are significant at *p* < 0.05 for Debre Markos (*gray dashed line*; t-stat = 3.9, *p* = 0.00057, *df* = 29) and Rob Gebeya (*black dashed line*; t-stat = 3.6, *p* = 0.0011, *df* = 29) and at *p* < 0.1 for Kurar (solid light gray line; t-stat = 1.89, *p* = 0.074, *df* = 21) using a Student’s t-test for linear trends over the period of record. For all three stations the slope of the trendline is ~0.3 °C per decade. The same statistical analysis indicated that there are no statistically significant linear trends for data shown in (**b**)

In terms of temperature changes, about 85 % of the respondents perceived that the temperature has increased over the last 20 years. This perception is significantly different from zero in all agro-ecosystems, and is also is significantly different, at a 95 % confidence level, in its intensity between agro-ecosystems.^1^ The strongest impression of increasing temperatures is in AES1 and AES3, 95 %, which is significantly greater (at a 95 % level) than in the other three zones, which are not statistically different from each other. This perception is consistent with scientific temperature measurements for Choke Mountain, which show linear trends in average monthly maximum temperature on the order of 0.03 °C per year, or ~0.3 °C per decade, since 1979, at stations located at low elevation, medium elevation, and high elevation (Fig. 3a).

Perceptions of precipitation change, with an average value of 61.3 % perceiving a decrease in rainfall, however, differed less significantly across agro-ecological zones. Only AES 3 with 55 % seeing a decrease in rainfall is significantly different at a 95 % level from AES 1 and AES 2, while AES 4 and AES 5 are not statistically significantly different from any of the other zones. The big difference to note is that in AES 3 relative to the other agro-ecosystems a much greater percentage, 31 %, thought that there was a change in the timing (seasonality) of the rainfall rather than a decrease in rainfall. In terms of extreme weather events, 85.8 % of farmers claimed to have been hit by an “extreme weather event or hazard related to climate change”, with the AES 5 zone showing the highest level at 95.6 %, which was statistically greater than all the other zones except AES 4. This shows the farmers at the highest altitudes being more vulnerable to extreme weather events than those at lower altitudes, AES 1, 2 and 3.

**Fig. 4:**
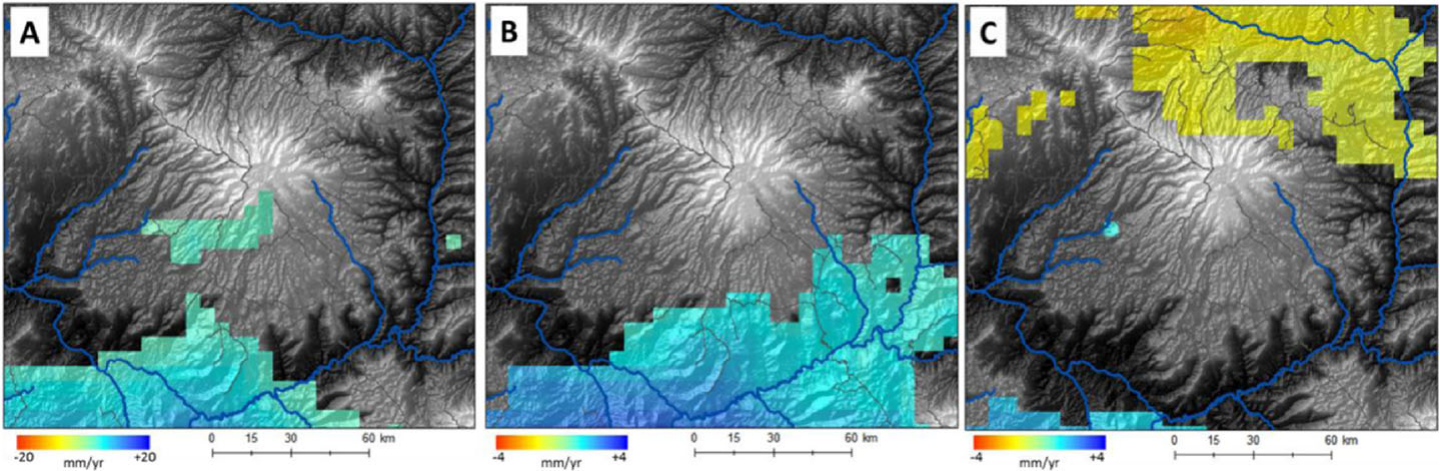
Trends in precipitation calculated for the period 1981–2012 using the USGS Climate Hazard Group InfraRed Precipitation with Station (CHIRPS) merged gauge and satellite product (Funk et al. 2013). Only trends significant at *p* < 0.1 (two-tailed student’s t-test for linear trend, *df* = 31) are shown for **a** annual, **b** June, and **c** September precipitation. *Gray shading* shows topography

These responses from farmer perceptions show that across all agro-ecosystems farmers on average perceive an increase in temperatures and decreases in precipitation. The relatively wide variation in perceptions across agro-ecosystems is understandable given the significant temporal and spatial variability in precipitation on Choke Mountain. It is interesting that a plurality of farmers perceived a decrease in precipitation when most measurements suggest no change or a small increase. In fact trends in precipitation are not statistically significant over most of the mountain (Fig. 4a; significance calculated using 2-tailed Student’s t-test for linear trend). Some changes in timing—an increase in early rainy season precipitation on southern slopes of the mountain and a reduction in late season precipitation at the end of the rainy season (Figs. 4b, c) have been observed, however, which is a trend identified by about a quarter of the farmers. As indicated in all panels of Fig. 4, objectively measured trends in precipitation total and timing have differed for different parts of the Mountain. These farmer perceptions being at odds with a number of the objective measures might reflect a climatic signal—if precipitation is steady but temperature rises, water stress may increase, thus giving the perception of a precipitation shortfall—or it might reflect a tendency to attribute a range of hardships relating to land use pressure, land degradation, and economic pressures to a lack of rain.

### Sensitivity profiles: ecosystems and agriculture

In previous studies we have reported on the agricultural suitability (Simane et al. 2012) and biophysical constraints on productivity (Simane et al. 2013) for each of the Choke Mountain AES. These studies found highest agricultural potential in the midland AES (AES 2 and AES 3), which are characterized by relatively gentle slopes and productive soils. Suitability is lower in AES 4 and AES 1, which are affected by acid, low fertility soils, and steep slopes with shallow soils, repsectively. AES 5 has very low suitability due to a combination of steep slopes and shallow, low fertility soils. Land degradation due to erosion is greatest in AES 1 and AES 5 and is moderately high in AES 4. The primary causes of enhanced erosion in these AES are deforestation and the use of traditional ox-drawn plows on steep slopes.

Land degradation has become one of the most important environmental problems on Choke Mountain, mainly due to soil erosion and nutrient depletion. Coupled with poverty and the fast-growing population, land degradation poses a serious threat to national and household food security. In order to reduce soil erosion, a number of soil conservation technologies have been introduced including physical measures (terracing and small check dams), fallowing, and plowing horizontally to the slope. Physical soil and water conservation (SWC) measures have been exercised by 34.9 % of the respondents (Table 4), with some significant variations between zones (AES 1 at 46 % is significantly higher than AES 2 at 25 %). However, efficiency of soil conservation structures (SCS) has been low in high rainfall areas of Ethiopia mainly due to crop yield reductions, increased soil erosion following breaching of SCS, and incompatibility with the tradition of cross-plowing and water-logging behind SCS (Simane 2011). Meanwhile the use of fallowing and avoiding crisscross plowing have been taken up by only a small minority of farmers, do not show much variation between agro-ecosystems and is therefore not included in the LVI-IPCC index.

**Table 4:** Land management practices and land fertility status, as reported in household surveys. All numbers are percent of responses within the given AES

	Measure	AES1	AES2	AES3	AES4	AES5	All households
Sustainable land management	Physical SWC	46.4	25.0	37.8	36.0	36.3	34.9
Significantly different (95 % level) from AES…		2	1	None	None	None	
Land productivity	Increased	39.5	37.6	32.0	24.3	18.0	33.3
	Decreased	55.6	59.4	63.3	62.1	75.3	62.6
	No change	4.9	2.9	3.6	6.7	6.7	4.1
Significantly different (95 % level) from AES…		5	5	5	5	1,2,3,4	

Table 4 also shows that farmers generally perceived decreased productivity on their farmlands. While 33 % of the total population has reported that land productivity has increased, the majority, 63 %, have reported that land productivity has decreased over the last 20 years due to land degradation, rainfall variability, drought and pests. The largest decrease in perceived land productivity, at 75 %, is in AES5, which is statistically different than AES 1, 2, and 4, while AES 3 falls in between the other zones and is not statistically different from them.

### Adaptive capacity profiles: wealth, technology, infrastructure, community and social capital

As shown in Table 5, across all five AES, the average land holding is 1.2 ha, with 94.3 % of the farmers owning their land. Among respondents, 50 % report that their land holding size has decreased over the past 20 years for reasons that include redistribution and land degradation, while 9 % have reported an increase in agriculturally useable land holdings as a result of clearing forest lands and grazing areas and redistribution of land. AES 3 and AES 4 stand out as having statistically significantly larger average land holding size, although the difference of a tenth of a hectare is small. In all agro-ecosystems farm sizes decreased on the majority of farms in all except AES 3 where only a third of the farms decreased in size. None of these differences in farmland changes, however, were statistically significantly different.

**Table 5:** Land and livestock holdings for each agro-ecosystem, as reported in household surveys

		AES1	AES2	AES3	AES4	AES5	Average
Land ownership (not significantly different)		95.2	91.3	97. 3	93.7	95.6	94.3
Average land holding size	(ha)	1.0	0.97	1.23	1.28	1.1	1.15
Significantly different (95 % level) from AES…		4	3, 4	2	1, 2	None	
Changes in farmland size over the last 20 years (not significantly different)	Increased	11.1	4.0	11.6	12.6	10.0	8.8
	Decreased	58	52.5	36.3	55	61.1	48.8
	No change	30.9	43.5	52.1	32.4	28.9	42.4
Possession of livestock	TLU	1.26	1.56	1.13	2.16	1.45	1.63
Significantly different (95 % level) from AES…		4	4	4, 2	1, 2, 3, 5	4	

All figures are % of farmers within the given AES, except for average land holding size which is hectares

*TLU* Tropical Livestock Unit

In quantifying livestock resources we aggregated all animals owned by farm households using tropical livestock units (TLU) (Jahnke and Asemanew 1983), and find that AES 4 has a statistically significantly higher level of livestock ownership than all other zones. The differences between AES 4 and the others imply major differences in livestock holdings and therefore wealth levels. For example, the difference in tropical livestock units between AES 4’s, 2.2 TLUs and AES 1’s 1.3 TLUs is the equivalent of a household in AES 4 having an extra two sheep and one cow. Households in the midland AES 3 also have significantly fewer livestock than AES 2 and 4, due in part to land pressures.

Differences in technology profiles between AES were attributed primarily to differences in the use of chemical fertilizer and improved seed as well as irrigation potential. The percentage of farmers who have applied chemical fertilizers across all AES in the Choke mountain area is high by Sub-Saharan Africa standards, with an average of 78 % having used chemical fertilizer. There exists, however, statistically significant differences between agro-ecosystems in fertilizer use with AES 5 at 50 % statistically significantly lower than all the other AES except for AES 1, which is at 64 %. Fertilizer use is correlated with livestock ownership (0.148, *p*-value = 0.000), suggesting that access to sources of wealth partially constrains farmers from using fertilizer. In addition, the total amount of chemical fertilizer applied is low, even among farmers that report using some fertilizer.

Irrigation use, the percentage of farmers with some irrigation on their land, varies between agro-ecosystems with an average of 20 % of the farms having some irrigation, while AES2’s 10 % average is statistically significantly below the overall average and that of AES3, 4, and 5. This difference primarily reflects drainage characteristics, as AES2 soils are prone to water-logging; irrigation could be useful in targeted seasons and years, but without adequate investment in drainage it is unlikely to be economically effective. On average, 16 % of farmers use improved seeds, with significant variation across agro-ecosystems. AES 5 has a statistically significantly lower level of improved seed use, 4 %, than all other zones. Meanwhile AES 1, 2 and 4 with rates between 19 and 22 % are statistically significantly greater than the rate of improved seed use in AES 3 of 10 %. In general, use of improved varieties in the midland agro ecologies of cool sub-moist mid-highlands, cool moist mid-highlands, and temperate moist mid-highlands is higher than in the extreme very cold sub-moist mid-highlands.

Access to major indicators of infrastructure (i.e., access to road, first cycle primary school, veterinary services, market, credit institution, electricity, and telephone; Table 6) are comparable to national average figures and do not vary significantly across agro-ecosystems. The majority of the human capital profile measures were provided in Table 2. In addition it is worth noting that 99 % of the households have access to agricultural extension and health services within 5 km of their homes a figure that does not vary significantly across agro-ecosystems. Social capital measures varied little and without statistical significance between agro-ecosystem and are omitted for brevity.

**Table 6:** Access to major indicators of infrastructure within 5 km of the home, as reported in household surveys

Indicator	Proportion of farmers with access (%)
Road	63
Primary school	99
Veterinary services	73
Market	95
Credit institutions	85
Electricity	42
Telephone	65

## Livelihood vulnerability index

Combining the measures in Table 1 using the methodology in Eqs. (1)–(5) provides a measure of the LVI-IPCC index for the five agro-ecosystems in the Choke mountain area. The results produce measures of exposure, sensitivity, and adaptive capacity, which all differ systematically across AES (Table 7). As is evident from the calculation of LVI-IPCC (Eq. 5), high values of exposure relative to adaptive capacity yield positive vulnerability scores while low values of exposure relative to adaptive capacity yield negative vulnerability scores. Sensitivity acts as a multiplier, such that high sensitivity in an AES for which exposure exceeds adaptive capacity will result in a large positive (i.e., high vulnerability) LVI-IPCC score. For Choke Mountain, exposure is high and adaptive capacity low for AES 1 and AES 5. This results in positive LVI-IPCC scores, which we classify here as “highly vulnerable” since it indicates an adaptation capacity deficit and high exposure relative to other AES. The opposite is true for AES 2 and AES 3, in which adaptive capacity exceeds exposure and overall vulnerability is deemed to be low. AES 4 exhibits intermediate vulnerability: the LVI-IPCC score is positive, but it is close to zero. The wealth, technology and infrastructure profiles are strong determinants of vulnerability. The community profile was another determinant, but, with fewer differences amongst the different agro-ecosystems, it played a secondary role. The social profile was not a major factor as there were few significant differences amongst the agro-ecosystems.

**Table 7:** Calculated indices for contributing factors and the Livelihood Vulnerability Index under the LVI-IPCC framework

	Exposure	Sensitivity	Adaptive capacity	LVI-IPCC
AES 1	0.78	0.30	0.21	0.72
AES 2	0.30	1.26	0.73	−0.62
AES 3	0.30	1.31	0.78	−0.72
AES 4	0.60	0.94	0.45	0.18
AES 5	0.76	0.21	0.25	0.71
Average	0.55	0.80	0.48	0.05

**Fig. 5:**
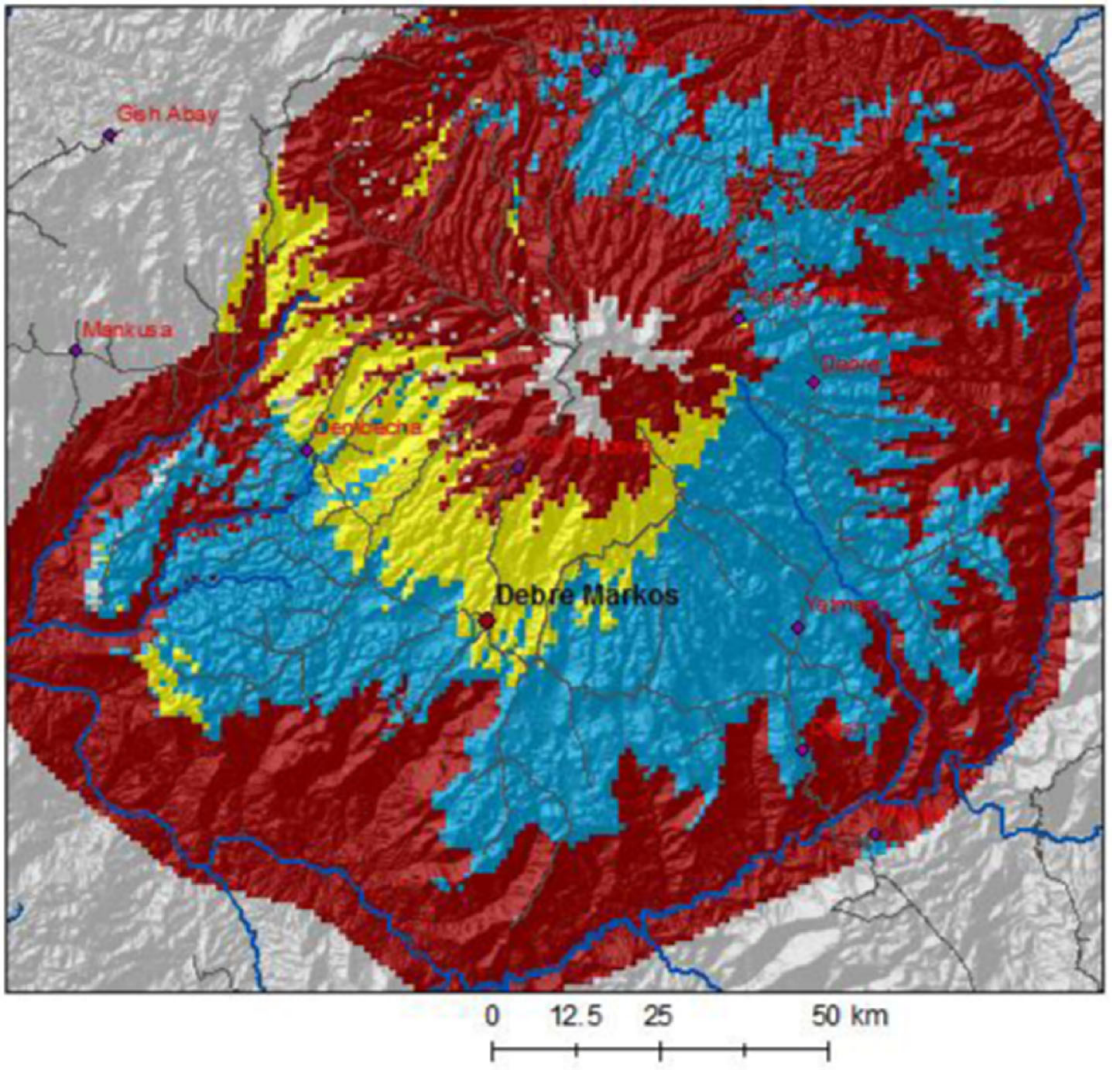
Relative Vulnerability Index for each AES on Choke Mountain. *Red* = highly vulnerable; *yellow* = moderately vulnerable; *blue* = less vulnerable. The map is cropped at the Blue Nile gorge to avoid extrapolation to areas that were not included in household survey. AES 6, the mountain summit, is also excluded from analysis

Put in geographic context, these results of estimating the LVI-IPCC index suggest that 30 % of the total land mass (midland plains with black soil (AES 2) and midland plains with brown soils (AES 3)) has relatively low vulnerability to climate change (Fig. 5). Overall, 62 % of the total land mass is categorized as having high relative vulnerability. This includes the lowland, valley fragmented AES 1 and the mountainous highland AES 5. With 8 % of the land mass—the midland sloping lands (AES 4)—is moderately vulnerable. The mountain summit is not classified on this map because there is no permanent population for LVI analysis, but it is known to have low agricultural potential and high susceptibility to overgrazing and deforestation (Simane et al. 2013). As this AES is largely uninhabited, however, it was not possible to include it in the formal LVI-IPCC analysis.

## Conclusions and recommendations

The LVI-IPCC offers a framework to evaluate and understand relative climate change vulnerability at household to community level in the Choke Mountain area, located in the Blue Nile Highlands region of Ethiopia. As this mountainous region is characterized by significant physiographic and climatic diversity, we applied LVI-IPCC at the scale of the agroecological system (AES)—a unit of aggregation that captures much of the physical, agricultural, and social diversity of the mountain. For Choke Mountain, the LVI-IPCC analysis indicated that lowland and the extreme highland AES have the highest perceived exposure to climate stresses and the most limited adaptive capability. This combination makes communities in these AES particularly vulnerable to climate change. Midland AES, in contrast, have lower perceived exposure and greater adaptive capacity, such that their climate vulnerability is relatively low. The process of performing the LVI-IPCC revealed a number of farmer-perceived constraints on productivity and stresses related to changing environmental conditions, which is useful for interventions in these low vulnerability but still relatively poor areas. For the most vulnerable AES the LVI-IPCC identifies particular adaptation barriers that can be targeted in future interventions, specifically lack of access to new technologies in the most vulnerable agro-ecosystems. Interestingly, basic infrastructure, education, access to markets, and extension services did not differ across AES, suggesting that the infrastructure backbone required to improve adaptive capacity is evenly distributed for the majority of communities on Choke Mountain.

The LVI-IPCC vulnerability analysis of Choke Mountain is now being used to inform community-driven climate resilience building strategies at “Climate Innovation Platforms” that have been established in select communities in all AES (Simane et al. 2012). Vulnerability analysis was an essential step in this process, as effective adaptation must be based on a solid understanding of local vulnerability, including adaptive capacity alongside exposure and sensitivity. This is an important consideration as the Climate Innovation Platforms model piloted on Choke Mountain is extended to other parts of Ethiopia, and perhaps beyond.

As with any generalization, AES-based analysis necessarily simplifies intra-community and temporal variability in vulnerability profiles (Smit and Wandel 2006; Swanson et al. 2009). Nevertheless, the systematic differences in LVI-IPCC observed between AES do indicate that the AES captures meaningful differences in vulnerability across the landscape of the study region. The method could be usefully applied to study landscape-scale vulnerability patterns across other tropical highland regions in a comparative perspective.

Finally, we emphasize that climate change is only one of the many challenges facing subsistence agricultural communities of Choke Mountain. Therefore, climate change adaptation must be advanced as a component of a holistic effort to build resilience of communities to the range of shocks and stresses that they experience. In this regard Choke Mountain is typical of subsistence agricultural regions throughout the tropics, and the LVI-IPCC analysis is best understood as a climate-oriented lens directed at the broader challenge of sustainable development.

## Appendix: household survey (English translation)

### Household survey

#### Introduction

This study is being carried out by Addis Ababa, John Hopkins and Wisconsin Universities to collect basic information/data about Community-based Innovation Platforms (CIPs) in the Choke Mountains’ watersheds and assess the problems of the surrounding communities to plan for sustainable adaptation and development activities.

The objective of this questionnaire is to collect primary data on socio-economics, ecologicy, and development that are required to assess the sustainability and effectiveness of established CIPs. Therefore, you are kindly requested to give your response freely and accurately to ensure the success of this study.

Dear respondents: You should be confident that the data/information which you give us works only for this study and the development of the target community.

Lastly, I thank you for your cooperation

## Footnotes

1 Here and in future uses of the term “significantly different” in this document we mean that a statistical test of difference in means is significant at a 95 % confidence level for a two tailed t-test.
